# Risky Decision Making in Juvenile Myoclonic Epilepsy

**DOI:** 10.3389/fneur.2018.00195

**Published:** 2018-03-26

**Authors:** Iris Unterberger, Laura Zamarian, Manuela Prieschl, Melanie Bergmann, Gerald Walser, Gerhard Luef, Andrija Javor, Gerhard Ransmayr, Margarete Delazer

**Affiliations:** ^1^Department of Neurology, Medical University of Innsbruck, Innsbruck, Austria; ^2^Department of Neurology 2, Kepler University Hospital, Linz, Austria

**Keywords:** juvenile myoclonic epilepsy, gambling, risk-taking, neuropsychology, cognition, executive functions

## Abstract

It is not known whether patients with juvenile myoclonic epilepsy (JME) differ from healthy people in decision making under risk, i.e., when the decision-making context offers explicit information about options, probabilities, and consequences already from the beginning. In this study, we adopted the Game of Dice Task-Double to investigate decision making under risk in a group of 36 patients with JME (mean age 25.25/SD 5.29 years) and a group of 38 healthy controls (mean age 26.03/SD 4.84 years). Participants also underwent a comprehensive neuropsychological assessment focused on frontal executive functions. Significant group differences were found in tests of psychomotor speed and divided attention, with the patients scoring lower than the controls. Importantly, patients made risky decisions more frequently than controls. In the patient group, poor decision making was associated with poor executive control, poor response inhibition, and a short interval since the last seizure episode. Executive control and response inhibition could predict 42% of variance in the frequency of risky decisions. This study indicates that patients with JME with poorer executive functions are more likely to make risky decisions than healthy controls. Decision making under risk is of major importance in every-day life, especially with regard to treatment decisions and adherence to long-term medical therapy. Since even a single disadvantageous decision may have long-lasting consequences, this finding is of high relevance.

## Introduction

Juvenile myoclonic epilepsy (JME) is the most common idiopathic generalized epilepsy syndrome, accounting for up to 12% of all epilepsies (1), with seizures typically manifesting around adolescence (2–4). Behavioral disturbances and psychiatric disorders have been reported to occur in approximately one third of patients with JME (5, 6), despite normal intelligence and unremarkable routine brain imaging (7). Functional and microstructural changes in the medial and dorsolateral prefrontal cortex and related subcortical structures have been recently reported for patients with JME by adopting advanced brain imaging methods (5–10). Correspondingly, several studies have suggested a distinct cognitive profile in JME, resembling predominantly frontal lobe dysfunction with disturbances in attention and executive functions (11–14). Recent studies have also shown that patients with JME perform lower than healthy controls when the decision-making context is (at least initially) ambiguous and learning from feedback is required (10, 14). So far, it is not known whether patients with JME would differ from healthy people in decision making under risk, i.e., when the decision-making context offers explicit information about options, probabilities, and consequences from the beginning, and optimal decisions can be estimated or also computed. This type of decision is of major importance in every-day life, especially with regard to treatment decisions and adherence to long-term medical therapy. The Game of Dice Task-Double (GDT-D) (15), a modified version of the GDT (16), is a computerized gambling task assessing decision making under risk. As executive functions play a major role in decision making under risk (17, 18), it could be hypothesized that patients with JME and lower executive functions tend to make more risky decisions compared to healthy controls. Difficulties in risk-related decision making are associated with lower quality of life and may even lead to long-lasting, negative consequences (19). An exact characterization of the problems encountered by this younger patient group is therefore highly relevant.

In this study, we aimed to investigate decision making under risk, when full information about options and consequences is provided, in both patients with JME and healthy controls which to our knowledge has not been done before. Furthermore, we wanted to examine the relationship between decision making under risk, neuropsychological performance, and clinical characteristics of patients with JME.

## Materials and Methods

### Study Collective

A total of 36 consecutive patients with classical JME were recruited at the outpatient epilepsy clinic of Medical University of Innsbruck between July 2013 and July 2016. The diagnosis of JME was based on the criteria of the International League against Epilepsy (2). Patients with epilepsy were compared to 38 gender-, education-, and age-matched (±2.5 years) healthy controls. These controls were recruited either from non-related acquaintances of patients or from hospital staff. All participants had normal intelligence (see Table 1) and had no history of neurological, psychiatric, and physical illnesses, except for epilepsy in the patient group. Moreover, participants did not take any other medication than antiepileptic drugs (AEDs) in the patient group. Patients did not have any types of seizure 1 week prior to study inclusion. A routine electroencephalography was performed in all patients at the day of study inclusion to exclude ictal discharges.

**Table 1:** Sample description.

	JME (*n* = 36)	HC (*n* = 38)
Gender, F/M	21/15	19/19
Age (years), mean (SD)	25.25 (5.29)	26.03 (4.87)
Education (years), mean (SD)	13.32 (2.69)	13.88 (2.29)
Estimated verbal intelligence quotient, mean (SD)	101.61 (11.24)	104.26 (10.90)

*JME, juvenile myoclonic epilepsy; HC, healthy controls*.

### Neuropsychological Background Assessment

All participants performed tests of psychomotor speed [Trail Making Test-A (TMT-A)] (20), cognitive flexibility (TMT-B) (20), categorical verbal fluency [animals/min; Regensburger Wortflüssigkeits-Test (RWT)] (21), phonological verbal fluency (s-words/min; RWT) (21), divided attention [subtest of Tests of Attentional Performance (TAP)] (22), response inhibition (TAP) (22), executive control (TAP) (22), and set-shifting (TAP) (22). They also responded to an inventory of sensation seeking (Arnett Inventory of Sensation Seeking) (23), to a multiple-choice test of estimated verbal intelligence (Mehrfachwahl Wortschatz Intelligenztest) (24), and to a self-rated questionnaire on anxiety and depression symptoms (Hospital Anxiety and Depression Scale—German version) (25).

### Decision-Making Task

We tested decision making under risk by means of the GDT-D (15), a modified version of the GDT (16). The GDT is a computerized gambling task, where participants have to maximize their fictitious starting capital of €1000 within 18 throws of a single virtual die. Participants are asked to guess which number will be thrown next by selecting one of the presented answer alternatives: single numbers (e.g., 3) or combinations of two (e.g., 1 2), three (e.g., 1 2 3), or four numbers (e.g., 1 2 3 4). Each alternative is associated with a specific winning probability and a specific amount of gain/loss. For example, the single-number alternative is associated with a winning probability of 1/6 and a gain/loss of €1000. By choosing the single-number alternative, the participant can win €1000 if the selected number is thrown; otherwise, s/he loses €1000. Alternatives and associated gains/losses remain visible on the computer screen for the whole task duration. Before beginning the task, participants are explicitly instructed about the rules and the amount of gains/losses associated with each alternative but not about which alternative is the most advantageous. After a choice is made, the computer indicates which number is thrown, whether the participant has won/lost, the residual capital, and the number of remaining throws (16). The GDT-D differs from the original task (16) in that participants are asked, after they make a choice but before they receive a feedback, whether they want to double their potential gain/loss. In the GDT-D, participants decide optimally when they choose to double their potential gain/loss after selecting the four-numbers alternative. Conversely, doubling the potential gain/loss after selecting the single-number alternative is the most risky choice. The GDT-D allows for the distinguishment between optimal choices and conservative choices as well as between risky decisions and non-risky decisions.

### Task Evaluation

We computed the net score by subtracting the number of high-risk choices (selection of single number and two-numbers combinations) from the number of low-risk choices (selection of three-numbers combinations and four-numbers combinations). A positive net score indicates a low-risk performance (advantageous behavior), whereas a negative net score indicates a high-risk performance (disadvantageous behavior). Furthermore, we analyzed how often participants selected each alternative and how often they decided to double the potential gain/loss (overall as well as after selection of the single-number alternative and the four-numbers alternative). Doubling the single-number alternatives is of special importance as it is extremely disadvantageous. Conversely, doubling the four-numbers alternatives can be considered an optimal decision. We also compared groups with respect to the mean expected value (MEV). The expected value for each single decision was calculated as follows: [(gain × winning probability) − (loss × losing probability)].

### Statistical Analysis

Statistical analysis was performed using SPSS 24.0 for Windows. The gender distribution was examined by χ^2^-test. Group comparisons in age, education, and estimated verbal intelligence were assessed using Student’s *t*-tests. Group differences in neuropsychological tests and in the GDT-D were investigated using multivariate analyses of variance (MANOVAs). With regard to latency variables, we submitted log-transformed reaction times to analysis. Tables report untransformed data. A Pearson correlation analysis was performed for each group separately between measures of decision making (no. of single-number selections, no. of four-numbers selections, net score, MEV, no. of optimal decisions, and total no. of doubling choices) and neuropsychological variables (categorical verbal fluency, phonological verbal fluency, psychomotor speed, cognitive flexibility, divided attention errors and omissions, response inhibition errors and omissions, executive control errors and omissions, set-shifting errors, intensity seeking, novelty seeking, anxiety, and depression). A correlation analysis was also performed for the patient group between decision making, demographical variables (age and education), and clinical variables (epilepsy duration, age at epilepsy onset, seizure frequency, and time since last seizure in months). For the patient group, we also performed a stepwise regression analysis to investigate which of the neuropsychological variables showing a significant correlation with the GDT-D could predict the patients’ decision-making performance. Significance was set at α = 0.05.

## Results

### Demographic and Clinical Characteristics of the Study Participants

A total of 36 epilepsy patients with a mean age of 25.25 years (SD 2.7) and a mean age at epilepsy onset of 14.28 years (SD 3.4) were included in this study. Patients were compared to 38 gender-, education-, and age-matched controls. Table 1 summarizes the demographic characteristics of the study population. Groups did not differ from each other in terms of age, years of formal education, and gender distribution. They were also comparable in terms of mean estimated verbal intelligence.

All but four patients (88.9%) were under current AED therapy (84.4% monotherapy, 15.6% polytherapy). The three most commonly used drugs were levetiracetam (20 patients), valproic acid (11 patients), and lamotrigine (4 patients). Epilepsy was well controlled in the majority of patients. Overall, during the last year, 21 out of 36 patients (58.4%) were completely seizure free. A more detailed patient description can be found in the Table S1 in Supplementary Material. We did not find any significant differences between seizure-free patients and non-seizure-free patients with regard to demographical, neuropsychological, and decision-making variables (see Table S1 in Supplementary Material).

### Neuropsychological Background Tests

Descriptive statistics and results of the MANOVA are presented in Table 2. In all tests, group scores were in the average range (16–84 percentiles) of standardized published norms (20–25). Group differences were significant in tests of psychomotor speed and divided attention, with the patients scoring lower than the controls. Group differences in other tests were not significant.

**Table 2:** Neuropsychological background assessment.

	JME (*n* = 36)		HC (*n* = 38)				
	Mean	SD	Mean	SD	Effect size *d*	*F* value	*p*
Overall multivariate analyses of variance						***F*_16,57_ = 1.98**	**0.030**
Categorical verbal fluency (test score)	24.56	6.29	26.24	5.80	−0.28	*F*_1,72_ = 1.43	0.236
Phonological verbal fluency (test score)	16.94	5.14	15.76	4.87	0.24	*F*_1,72_ = 1.03	0.313
Psychomotor speed (time in seconds)	30.92	18.71	22.66	6.31	**0.59**	***F*_1,72_ = 8.34**	**0.005**
Cognitive flexibility (time in seconds)	61.11	28.21	58.61	13.73	0.11	*F*_1,72_ = 0.06	0.809
Divided attention (time in milliseconds)^a^	698.31	100.83	651.70	51.36	**0.58**	***F*_1,72_ = 6.04**	**0.016**
Divided attention (errors and omissions, sum)	4.92	3.95	3.24	3.08	**0.47**	***F*_1,72_ = 4.18**	**0.044**
Response inhibition (time in milliseconds)	395.28	74.58	365.71	52.48	0.46	*F*_1,72_ = 3.57	0.063
Response inhibition (errors and omissions, sum)	1.81	2.89	1.18	1.11	0.29	*F*_1,72_ = 1.52	0.221
Executive control (time in milliseconds)	606.58	98.12	572.63	78.15	0.38	*F*_1,72_ = 2.38	0.127
Executive control (errors and omissions, sum)	3.81	3.96	2.66	2.37	0.35	*F*_1,72_ = 2.32	0.132
Set-shifting (time in milliseconds)	683.03	234.12	599.29	109.70	0.46	*F*_1,72_ = 3.55	0.063
Set-shifting (errors)	2.83	3.11	2.66	2.11	0.06	*F*_1,72_ = 0.08	0.776
Intensity seeking (test score)	26.61	4.38	27.16	4.06	−0.13	*F*_1,72_ = 0.31	0.579
Novelty seeking (test score)	24.58	3.79	25.26	4.85	−0.16	*F*_1,72_ = 0.45	0.505
Anxiety symptoms (test score)	5.11	2.66	4.47	3.62	0.20	*F*_1,72_ = 0.74	0.393
Depression symptoms (test score)	2.58	2.88	2.84	2.94	−0.09	*F*_1,72_ = 0.15	0.703

*Bold values indicate statistical significance (*p* < 0.05)*.

*JME, Juvenile myoclonic epilepsy; HC, healthy controls*.

*^a^We computed a mean reaction time measure between visual and auditory conditions*.

### Decision-Making Task (GDT-D)

Descriptive statistics and results of the MANOVA are presented in Table 3. Since only few participants decided to double their win/loss with the single-number alternative (extremely risky decisions; JME: 5/36, 13.89%; HC: 5/38, 13.16%), we did not include this variable into the analysis. Results of the MANOVA indicated a significant difference in how frequent groups selected the single-number alternative, with the patients making risky choices more often than controls. Group differences were not significant in other measures of decision making.

**Table 3:** Game of dice task-double.

	JME (*n* = 36)		HC (*n* = 38)				
	Mean	SD	Mean	SD	Effect size *d*	*F* value	*p*
Overall multivariate analyses of variance						***F*_6,67_ = 2.23**	**0.050**
Net score	8.39	7.72	10.37	6.91	−0.27	*F*_1,72_ = 1.35	0.249
Single numbers (no. of selections)	1.36	1.97	0.47	1.01	**0.57**	***F*_1,72_ = 6.03**	**0.016**
Two-numbers combinations (no. of selections)	3.44	2.58	3.34	3.01	0.04	*F*_1,72_ = 0.02	0.876
Three-numbers combinations (no. of selections)	7.14	4.20	8.00	3.26	−0.23	*F*_1,72_ = 0.98	0.327
Four-numbers combinations (no. of selections)	6.06	4.28	6.18	4.99	−0.02	*F*_1,72_ = 0.01	0.906
Doubling after selection of four-numbers combinations	3.14	3.20	3.45	4.07	−0.08	*F*_1,72_ = 0.13	0.719
Total no. of doubling choices	7.33	5.05	8.97	5.99	−0.30	*F*_1,72_ = 1.61	0.208
Mean expected value	−86.88	141.93	−52.34	102.96	−0.28	*F*_1,72_ = 1.45	0.233

*Bold values indicate statistical significance (*p* < 0.05)*.

*JME, Juvenile myoclonic epilepsy; HC, healthy controls*.

### Correlation Analysis

For the patient group, we found that poor decision-making performance was associated with poor executive control, poor response inhibition, and a short interval since the last seizure episode (see Table 4). Similarly, for the control group, we found that poor decision-making performance was related to poor executive control. Additionally, high frequency of doubling choices with all types of alternatives was associated with poor set-shifting.

**Table 4:** Pearson correlation analysis between game of dice task-double measures and neuropsychological/clinical variables.

	Single numbers (no. of selections)	Four-numbers combinations (no. of selections)	Net score	Mean expected value	Total no. of doubling choices
**JME**					
Executive control (errors and omissions, sum)	0.558***		−0.377*	−0.569***	
Response inhibition (errors and omissions, sum)	0.554***		−0.489**	−0.453**	
Time since last seizure (months)		0.335*			
**HC**					
Executive control (errors and omissions, sum)	0.567***		−0.371*		
Set-shifting (errors)					0.344*

*JME, Juvenile myoclonic epilepsy; HC, healthy controls*.

*p < 0.05, **p < 0.01, ***p < 0.001.

### Regression Analysis

A stepwise regression analysis was conducted to evaluate whether executive control and response inhibition would predict the patients’ performance on the GDT-D. As dependent variable we used the number of single-number selections since patients significantly differed from controls in this measure. At step 1 of the analysis, “Executive Control” entered into the regression equation and was significantly correlated with the dependent variable [*F*(1, 34) = 15.40, *p* < 0.001]. The multiple correlation coefficient was 0.31, indicating that approximately 29.1% of variance in the GDT-D could be accounted for by performance on the executive control test. At step 2 of the analysis, “Response Inhibition” was added into the equation and was significantly correlated with the dependent variable [*F*(2, 33) = 13.48, *p* < 0.001]. “Response Inhibition” explained significantly more variance [*R*^2^ change = 0.14; *F*(1, 33) = 8.27, *p* < 0.01]. The final multiple correlation coefficient was 0.45, indicating that approximately 41.6% of variance in the GDT-D (single-number choices) could be accounted for by both executive control and response inhibition. Table 5 gives additional information about the predictor variables included in the final statistical model.

**Table 5:** Unstandardized and standardized regression coefficients for the variables included in the model.

Variable	B	SE B	β
Executive controls (errors and omissions, sum)	0.20	0.07	0.41**
Response inhibition (errors and omissions, sum)	0.27	0.10	0.40**

**p < 0.01.

## Discussion

In this study, we compared performance of patients with JME to that of healthy controls on the GDT-D, a task of decision making under explicit risk conditions (information about options, probabilities, and consequences is explicitly given from the beginning) and on a comprehensive neuropsychological test battery.

Patients with JME scored significantly lower than healthy controls in tests of psychomotor speed and divided attention. This finding is in line with previous studies reporting predominantly frontal lobe dysfunction in patients with JME (11–14). In the GDT-D, patients scored at the same level as controls in several outcome measures (net score, MEV, and number of optimal decisions). However, patients made significantly more risky decisions than controls. Results of a correlation analysis for the patient group indicated that poor decision making was associated with poor executive control and response inhibition. To evaluate whether both executive control and response inhibition would predict the patients’ performance on the GDT-D, we also performed a stepwise regression analysis. Results indicated that 42% of variance in the number of risky selections could be predicted by these two measures of executive functions. The importance of executive functions in decision making under risk has been shown in previous studies, both in healthy participants and in patients with various neurological disorders (15, 26–29). As outlined by Schiebener et al. (30), not all executive functions contribute to advantageous decision making to the same extent. In a large study with healthy participants, three components were assessed, i.e., general cognitive self-control, concept formation, and monitoring. General cognitive self-control was identified to be the strongest predictor of decision making under risk and to mediate the effect of concept formation and monitoring. General cognitive self-control implies the ability to inhibit automatic responses which are not in accordance with the task’s rules and goals (30). With regard to performance on the decision-making task, cognitive self-control may be responsible for inhibiting the activation of a schema linked to unplanned choices or for inhibiting the impulse of choosing the option associated with the highest possible monetary gain. Cognitive self-control also enables individuals to apply a new strategy when the current strategy does not seem appropriate. Results of our study are in line with Schiebener et al. (30) as they point to the importance of response inhibition and executive control in avoiding disadvantageous, risky choices in the JME group. It should be noted, however, that executive functions account only for part of the variance found in decision making. Other cognitive abilities, including numerical abilities and ratio processing, either contribute directly to advantageous decisions or are mediated *via* executive functions (15, 31).

Our findings add to previous studies on decision making in patients with JME. While this study investigated decision making under risk with explicit information on task contingencies, previous studies on JME have focused on decision making under initial ambiguity adopting the Iowa Gambling Task (IGT) (10, 14). When the decision situation is ambiguous, probabilities and outcomes are, at least at the beginning of a task, unknown. However, feedback on previous decisions can be used to choose the most advantageous options. Conversely, in decisions under risk, the situation is exactly defined from the beginning. Also, options, probabilities, and consequences are explicitly given or can be estimated (15). Recent research has provided evidence that the neural signals vary between decision situations reflecting different degrees of uncertainty and that different brain circuits are involved in decisions under risk and in decisions under ambiguity (32, 33). In both studies adopting the IGT (10, 14), patients with JME showed difficulties in learning to choose advantageous options. Both studies also reported a correlation between executive functions and decision making. Poor performance on the IGT was associated with an increased activation in the dorsolateral prefrontal cortex during a functional magnetic resonance working memory task, emphasizing frontal lobe dysfunction in patients with JME (10). Our study adds to these findings suggesting that low executive control and response inhibition also predict disadvantageous decisions under exactly defined conditions.

In this study, the direct comparison of seizure-free patients and non-seizure-free patients did not yield any significant results. However, we found that poor decision making was associated with a short interval since the last seizure episode. These results add to previous investigations suggesting that problems in decision making are more pronounced in patients with ongoing seizures (14). The fact that we did not find any significant differences between seizure-free patients and non-seizure-free patients when directly compared to each other might be due to the small sizes of the two subgroups. Future studies should investigate the impact of disease-related variables in larger groups of patients with JME.

Another limitation of our study regards the lack of an additional group of patients taking the same medications but having a different type of epilepsy. Although cognitive side effects of AEDs are very often subtle (34), they may have an impact on complex cognitive mechanisms such as decision making. Indeed, previous investigations have shown that AEDs compared to nondrug conditions may impair performance in executive functions tasks (35). In this study, all but four patients (88.9%) were under AED therapy, and we cannot disentangle the possible influence of AED treatment from other disease-related conditions. As in other investigations (10, 14, 36–38), decision-making deficits were observed under current antiepileptic medication. However, a recent study on TLE and decision-making (39) indicates that AED are unlikely to be the major cause of risky decision making. In the study by Delazer et al. (39), two groups of patients (one with structural abnormalities in the mesial temporal lobe, the other with abnormalities in the lateral, basal, or polar parts of the temporal lobe) received standard AED therapy, but only the group with mesial temporal lobe epilepsy showed decision-making deficits. It should be noted, however, that Delazer et al. (39) used a different decision-making task (IGT) as the one adopted here (GDT-D). In our study, specific AED effects on cognition could not be explored due to the small sample sizes of different medications. Future studies might investigate the impact of AED therapy on decision making and risky behavior in more detail.

In conclusion, our findings show that the number of optimal decisions did not differ between patients and healthy controls. However, patients with JME made more risky decisions. High levels of executive control and response inhibition seem to be essential for making less risky decisions. Several cognitive models have been proposed where reflective processing, including executive control, guides decision making. Apart from reflective processing, heuristics, intuition, and emotions are crucial in the process of decision making. Recent studies propose that reflective processing and intuitive processing interact and inform each other and that advantageous decision-making relies on both, intuitions and reflections (18, 40, 41). While healthy people switch between resources without any effort and may safely follow their intuitions without hesitation, patients with low cognitive control may be attracted by risky, disadvantageous alternatives. Providing full and exact information (e.g., about treatment options and consequences) and encouraging deliberate and slow reasoning may lead to safe and advantageous decisions also in patients with JME. This may be of particular importance when discussing possible treatment options with patients.

## Ethics Statement

This study was carried out in accordance with the recommendations of the Declaration of Helsinki with written informed consent from all subjects. The protocol was approved by the research ethics committee at Innsbruck Medical University.

## Author Contributions

IU, LZ, AJ, GR, and MD generated the research idea and concept. IU, LZ, and MD were responsible for data acquisition and interpretation, literature review, and manuscript preparation. IU, LZ, MP, MB, GW, GL, AJ, GR, and MD made critical revisions for important intellectual content and approved the final manuscript.

## Conflict of Interest Statement

AJ has moved to Biogen International GmbH, Zug, Switzerland since completion of the work. None of the other authors has any conflict of interest to disclose.
