# Supplementary material for: Risky Decision Making in Juvenile Myoclonic Epilepsy
Source: Front Neurol. 2018 Mar 26;9:195. doi: 10.3389/fneur.2018.00195 (PMC5879545; doi:10.3389/fneur.2018.00195)
Supplement: Supplementary file 1 [file Table_1.PDF]

Supplementary Table 1. Detailed patient description.

	JME<br>(n = 36)	SF<br>(n = 21)	nSF<br>(n = 15)
**Age at epilepsy onset (years), mean (SD)**	14.28 (3.39)	14.57 (3.38)	13.87 (3.46)
**Duration of epilepsy (years), mean (SD)**	6.65 (4.56)	5.11 (3.41)	8.80 (5.17)
**Time since last seizure (months), mean (SD)**	35.72 (49.64)	59.10 (54.13)	3.00 (2.75)
**Generalized seizures, frequency (%)**			
**Absence, frequency (%)**	9 (25.00)	7 (33.33)	2 (13.33)
**Myoclonic, frequency (%)**	100 (100)	100 (100)	100 (100)
**Tonic-clonic, frequency (%)**	31 (86.11)	18 (85.71)	13 (86.66)
**Seizure frequency**			
**Seizure free > 1 year, frequency (%)**	21 (58.33)		
**0 / last 6 months, frequency (%)**	3 (20.00)		
**<1/month, frequency (%)**	2 (13.33)		
**1-5 / month, frequency (%)**	8 (53.33)		
**> 5-10 / month, frequency (%)**	2 (13.33)		
**Routine EEG (at time of investigation)**			
**Normal, frequency (%)**	24 (66.66)	13 (61.90)	11 (61.11)
**Generalized epileptic discharges, frequency (%)**	15 (41.66)	8 (38.09)	7 (38.88)
**Sleep deprivation, frequency (%)**	28 (77.77)	15 (71.42)	14 (93.33)
**Family history of epilepsy, frequency (%)**	13 (36.11)	8 (38.09)	5 (33.33)
**AED at time of the investigation**			
**None, frequency (%)**	4 (11.11)	3 (14.28)	1 (6.66)
**VPA, frequency (%)**	8 (22.22)	6 (28.57)	2 (13.33)
**LEV, frequency (%)**	16 (44.44)	9 (42.86)	7 (46.66)
**LTG, frequency (%)**	2 (5.55)	0 (0.00)	2 (13.33)
**TPM, frequency (%)**	1 (2.77)	0 (0.00)	1 (6.66)
**LEV + LTG, frequency (%)**	1 (2.77)	1 (4.76)	0 (0.00)
**LEV + PTM, frequency (%)**	1 (2.77)	1 (4.76)	0 (0.00)
**LEV + VPA, frequency (%)**	2 (5.55)	1 (4.76)	1 (6.66)
**VPA + LTG, frequency (%)**	1 (2.77)	0 (0.00)	1 (6.66)

Abbreviations: JME = patients with juvenile myoclonic epilepsy; SF = seizure-free patients; nSF = not seizure-free patients; GTCS = generalized tonic-clonic seizure; AED = antiepileptic drug; VPA = valproate; LEV = levetiracetam; LTG = lamotrigine; TPM = topiramate; PTM = petinimid.
